# Supplementary material for: Ionic Chiral Ferrocene Doped Cholesteric Liquid Crystal with Electronically Tunable Reflective Bandwidth performance
Source: Materials (Basel). 2022 Dec 8;15(24):8749. doi: 10.3390/ma15248749 (PMC9783301; doi:10.3390/ma15248749)
Supplement: Supplementary file 1 [file materials-15-08749-s001.zip › materials-2048229-supplementary.pdf]

Table of Contents

A series of ferrocene chiral ions (CD-Fc^+) were designed and prepared to prepare ferrocene chiral ion derivatives/liquid crystals with electronically tuneable reflection bandwidth. The experimental results showed that the reflective band of ionic ferrocene doped liquid crystals can be broadened after a certain electric field applied. Then after the reverse electric field applied, the reflection bandwidth can be restored to the initial state.

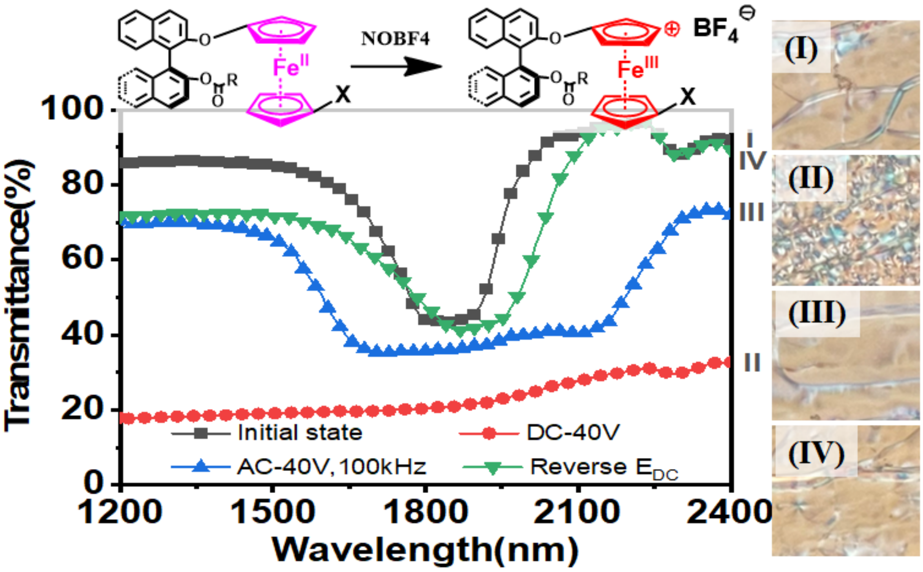

Electronic Supplementary Information

1 Synthesis

[1,1'-Binaphthyl]-2'-hydroxy-2-ylferrocene formate (CD-Fc1)

[1,1'-Binaphthyl]-2'-hydroxy-2-ylferrocene formate (CD-Fc1) was synthesized through the following conventional methods. The ferrocene carboxylic acid (5.0 g, 21.7 mmol) and (R)-(+)-1,1'-bin-2-naphthol ($[a]_D^{25}$ = +35.2°, 8.0 g, 27.9 mmol) were added to a 500 mL single-neck flask. In addition, the dehydrating agent 1-ethyl-3-(3-dimethylaminopropyl) carbodiimide (EDC, 8.5 g, 69.5 mmol) and catalytic amount of 4-dimethylaminopyridine (DMAP) were also placed in the flask. To completely dissolve the above-mentioned reactants, 150 mL dichloromethane was poured into the flask. The reaction was stirred at room temperature for 30.0 h. The product was observed with TLC plate by using solvent (dichloromethane/petroleum ether=3:1, Rf = 0.7). The product was extracted by column chromatography with dichloromethane: petroleum ether (3:1) as eluent, and the solvent was evaporated by a rotary evaporator to obtain 8.9 g. The yield was 82%. MALDI-TOF-MS m/z $[M]^+$: 497.983. FT-IR (KBr, cm-1): 3384 (-OH stretching), 1695 (-C=OC- stretching), 1597, 1510, 1450 (C=C stretching), 816 (πC-H bending). ^{1}H NMR (500 MHz, $CDCl_3$) δ (ppm) 8.10 (OH, d, J = 8.9 Hz, 1H), 8.0 (ArH, d, J = 8.2 Hz, 1H), 7.94 (ArH, d, J = 9.0 Hz , 1H), 7.89 – 7.84 (ArH, m, 1H), 7.53 (ArH, ddd, J = 8.1, 6.8, 1.2 Hz, 1H), 7.45 (ArH, dd, J = 15.2, 8.9 Hz, 2H), 7.36 (ArH, dddd, J = 12.0, 8.5, 6.7, 1.5 Hz, 3H), 7.23 (ArH, dddd, J = 15.3, 7.5, 2.2, 0.9 Hz, 2H), 5.70 – 5.62 (ArH, m, 1H), 4.70 (FcH, dt, J = 2.6, 1.3 Hz, 1H), 4.59 (FcH, dt, J = 2.5, 1.2 Hz, 1H), 4.34 (FcH, dd, J = 16.9, 2.6, 1.4 Hz, 2H), 3.65 (Fc-H, s, 5H).

[1,1'-Binaphthyl]-2'-((4-pentylcyclohexane-1-carbonyl)oxy)-2-ylferrocene carboxylate (CD-Fc-BipOC4)

[1,1'-Binaphthyl]-2'-((4-pentylcyclohexane-1-carbonyl)oxy)-2-ylferrocene carboxylate (CD-Fc-BipOC4) according to the conventional routine synthesis. The CD-Fc1 (1.0 g, 2.01 mmol) and achiral 4'-butoxy-[1,1'-biphenyl]-4-carboxylic acid (0.68 g, 2.5 mmol) were weighed and then added to a 250.0 mL single-neck round bottom flask. In addition, the dehydrating agent EDC (0.78g, 4.02mmol) and an appropriate amount of DMAP were also placed in the flask. To completely dissolve the above mentioned reactants, an appropriate amount of dichloromethane was poured into the flask. The reaction was stirred at room temperature for 30.0 h. The rotary evaporator was appointed to evaporate the solvent phase. The product was observed with TLC plate by using dichloromethane (Rf = 0.8). The product was extracted by column chromatography with dichloromethane, and the solvent was evaporated by a rotary evaporator to obtain CD-Fc-BipOC4 as 1.26g. The yield was 93%. MALDI-TOF-MS m/z $[M]^+$: 677.801. FT-IR (KBr, cm-1): 2953,2872(-CH_3 stretching), 2930,2850(-CH_2- stretching), 1730(-C=OC- stretching), 1592,1510,1450(C=C stretching)), 828 (πC-H bending). ^{1}H NMR (500 MHz, $CDCl_3$) δ (ppm) 8.03 (ArH, dd, J = 14.4, 8.9 Hz, 2H), 7.95 (ArH, t, J = 8.4 Hz, 2H), 7.56 – 7.43 (ArH, m, 5H), 7.36 (ArH, dddd, J = 36.0, 8.2, 6.7, 1.4 Hz, 2H), 7.20 (ArH, dd, J = 8.4, 1.1 Hz, 1H), 4.67 (FcH, d, J = 2.1 Hz, 1H), 4.56 (FcH, s, 1H), 4.30 (FcH, d, J = 9.5 Hz, 2H), 3.59 (FcH, s, 5H), 2.10 (-CO-CH-, ddt, J = 17.9, 12.8 , 4.4 Hz, 1H), 1.70 – 1.49 (-CH_2-, m, 2H), 1.49 – 1.04 (-CH_2-, m, 7H), 1.06 – 0.60 (-CH_2CH_3, m, 11H). Elemental analysis: Calcd. for $C_{48}H_{38}FeO_5$: C, 76.80; H, 5.10; Fe, 7.44; found: C 76.86%, H 5.19%; Fe 7.31%.

[1,1'-Binaphthyl]-2'-((4'-((2-ethylhexyl)oxy)-[1,1'-biphenyl]-4-carbonyl)oxy)-2- Ferrocene formate (CD-Fc-BipOC8)

The synthesis of [1,1'-Binaphthyl]-2'-((4'-((2-ethylhexyl)oxy)-[1,1'-biphenyl]-4-carbonyl)oxy)-2-Ferrocene formate (CD-Fc-BipOC8) was similar to the synthesis of **CD-Fc-BipOC4** by using racemic 4'-(octyloxy)-[1,1'-biphenyl]-4-carboxylic acid as the raw material. MALDI-TOF-MS m/z$[M]^+$: 805.924. FT-IR (KBr, cm-1): 2958, 2870 (-CH_3 stretching), 2926, 2858 (-CH_2- stretching), 1734 (-C=OC-stretching), 1603, 1499, 1451 (C=C stretching), 1184 (-COC- bending), 825 (πC-H bending). ^{1}H NMR (500 MHz, $CDCl_3$) δ (ppm) 8.12 (ArH, d, J = 8.9 Hz, 1H), 8.04 – 7.85 (ArH, m, 3H), 7.85 – 7.61 (ArH, m, 3H), 7.60 – 7.44 (ArH, m, 8H), 7.42 (ArH, ddd, J = 8.4, 6.7, 1.3 Hz, 1H), 7.38 – 7.29 (ArH, m, 2H), 7.05 – 6.85 (ArH, m, 2H), 4.76 – 4.66 (FcH, m, 1H), 4.65 – 4.54 (FcH, m, 1H), 4.32 (FcH, dtd, J = 12.3, 2.6, 1.3 Hz, 2H), 3.89 (-O-CH2-,dd, J = 5.7, 1.9 Hz, 2H), 3.62 (FcH, s, 5H), 1.83 – 1.63 (-CH-, m, 1H), 1.57 –

1.39 (-CH_2-, m, 4H), 1.38 – 1.30 (-CH_2 -, m, 4H), 0.97 (-CH_2-, s, 2H), 0.96 – 0.87 (-CH_3, m, 6H). Elemental analysis: Calcd. for $_{52}H_{46}FeO_5$: C, 77.42; H, 5.75; Fe, 6.92; found: C 77.56%, H 5.79%; Fe 6.85%.

[1,1'-Binaphthyl]-2'-((4-heptylbenzoyl)oxy)-2-ylferrocene carboxylate (CD-Fc-BenC7)

The specific synthesis routine of [1,1'-Binaphthyl]-2'-((4-heptylbenzoyl)oxy)-2-ylferrocene carboxylate (CD-Fc-BenC7) was similar to the synthesis of **CD-Fc-BipOC4** by using achiral 4-heptylbenzoic acid as the raw material. MALDI-TOF-MS m/z $[M]^+$: 700.261. FT-IR (KBr, cm-1): 2950, 2870 (-CH_3 stretching), 2926, 2853 (-CH_2- stretching), 1735 (-C=OC- stretching), 1610, 1510, 1450 (C=C stretching), 820 (πC-H bending). ^1H NMR (500 MHz, $CDCl_3$) δ (ppm) 8.10 (ArH, d, J = 8.9 Hz, 1H), 8.01 – 7.88 (ArH, m, 3H), 7.75 (ArH, d, J = 8.9 Hz, 1H) , 7.59 (ArH, d, J = 8.1 Hz, 2H), 7.53 – 7.44 (ArH, m, 4H), 7.41 (ArH, ddd, J = 8.4, 6.7, 1.3 Hz, 1H), 7.37 – 7.25 (ArH, m, 2H), 7.08 (ArH, d, J = 8.1 Hz, 2H), 4.75 – 4.66 (FcH, m, 1H), 4.59 (FcH, dd, J = 2.5, 1.3 Hz, 1H), 4.31 (FcH, ddd, J = 9.1, 2.5, 1.2 Hz, 2H), 3.61 (FcH, d, J = 1.0 Hz, 5H), 2.58 (Ar-H, t, J = 7.7 Hz, 2H), 1.28 (-CH_2-, dt, J = 10.8, 5.9 Hz, 10H), 0.90 (-CH_3, d, J = 6.6 Hz, 3H). Elemental analysis: Calcd. for $C_{45}H_{40}FeO_4$: C, 77.14; H, 5.75; Fe, 7.97; found: C 77.19%, H 5.79%; Fe 7.90%.

1'-((4-(4-propylcyclohexyl)phenoxy)carbonyl)ferrocenecarboxylic acid (Fc2)

1'-((4-(4-propylcyclohexyl)phenoxy)carbonyl) ferrocene carboxylic acid (Fc2) was synthesized through the conventional routine method. The 1,1'-ferrocene dicarboxylic acid (3.28 g, 12.0 mmol) and 4-(4-propylcyclohexyl)phenol (1.97 g, 8.0 mmol) were weighed and then added to a 250.0 mL single-neck round bottom flask. In addition, the dehydrating agent EDC (3.10g, 16.0mmol) and an appropriate amount of catalyst DMAP were also placed in the flask. To completely dissolve the above mentioned reactants, an appropriate amount of dichloromethane was poured into the flask. The reaction was stirred at a constant temperature of 30 °C for 36.0 h. The rotary evaporator was appointed to evaporate the solvent phase. The product was observed with TLC plate by using ethyl acetate (Rf = 0.4). The product was extracted by column chromatography with ethyl acetate, and the solvent was evaporated by a rotary evaporator to obtain 2.28g. The yield was 60%. MALDI-TOF-MS m/z$[M]^+$: 474.138. FT-IR (KBr, cm-1): 3120 (-COOH stretching), 2953, 2865 (-CH_3 stretching), 2920, 2850 (-CH_2- stretching), 1721 (-C=OC- stretching), 1507, 1456 (C=C stretching), 830 (πC-H bending). ^1H NMR (500 MHz, $CDCl_3$) δ (ppm) 7.27 (ArH, s, 1H), 7.15 (ArH, d, J = 8.0 Hz, 3H), 5.02 (FcH, t, J = 1.9 Hz, 2H), 4.96 (FcH, t, J = 1.9 Hz, 2H), 4.70 – 4.45 (FcH, m, 4H), 2.51 (Ar-H, t, J = 12.1 Hz, 1H), 1.91 (-CH_2-,s, 2H) , 1.88 – 1.42 (-CH_2-, m, 4H), 1.38 (-CH2-, dd, J = 15.8, 8.1 Hz, 2H), 1.32 – 1.16 (-CH-CH_2-, m, 3H), 1.08 (-CH_2-, q, J = 12.7, 12.1 Hz, 2H), 0.93 (-CH_3, t, J = 7.3 Hz, 3H).

[1,1'-Binaphthyl]-2'-hydroxy-2-yl-1'-((4-(4-propylcyclohexyl)phenoxy)carbonyl)ferrocene dicarboxylate (CD-Fc2)

The specific synthesis routine of [1,1'-Binaphthyl]-2'-hydroxy-2-yl-1'-((4-(4-propylcyclohexyl)phenoxy)carbonyl)ferrocene dicarboxylate (CD-Fc2) was referred to the synthesis method of CD-Fc mentioned before. MALDI-TOF-MS m/z$[M]^+$: 742.24. FT-IR (KBr, cm-1): 2954, 2868 (-CH_3 stretching), 2920, 2846 (-CH_2- stretching), 1733 (-C=OC- stretching), 1597, 1509, 1454 (C=C stretching), 815 (πC-H bending). ^1H NMR (400 MHz, $CDCl_3$) δ (ppm) 8.09 (-OH, d, J = 8.8 Hz, 1H), 7.97 (ArH, dd, J = 21.0, 8.6 Hz, 2H), 7.86 (ArH, dd, J = 8.0, 1.5 Hz, 1H), 7.59 – 7.46 (ArH, m, 2H), 7.46 – 7.31 (ArH, m, 4H), 7.31 – 7.17 (ArH, m, 4H), 7.12 – 6.98 (ArH, m, 2H), 5.56 (ArH, s, 1H), 4.72 (FcH, ddt, J = 16.5, 2.5, 1.3 Hz, 2H), 4.61 (FcH, dt, J = 2.6, 1.3 Hz, 1H), 4.45 (FcH, dp, J = 3.9, 1.4 Hz, 2H), 4.42 (FcH, dd, J = 2.6, 1.3 Hz, 1H), 3.80 (FcH, td, J = 2.6, 1.3 Hz, 1H), 3.67 (FcH, td, J = 2.6, 1.3 Hz, 1H), 2.63 – 2.36 (Ar-H, m, 1H), 2.03 – 1.78 (-CH_2-, m, 4H), 1.52 – 1.18 (-CH_2-CH-, m, 7H) , 1.06 (-CH_2-, qd, J = 13.2, 12.6, 3.8 Hz, 2H), 0.93 (-CH_3, d, J = 7.2 Hz, 3H).

[1,1'-Binaphthyl]-2'-(4-heptylbenzoyl)oxy-2-yl-1'-((4-(4-propylcyclohexyl)phenoxy)carbonyl) Ferrocene dicarboxylate (CD-Fc2-BenC7)

The specific synthesis routine of [1,1'-Binaphthyl]-2'-(4-heptylbenzoyl)oxy-2-yl-1'-((4-(4-propylcyclohexyl)phenoxy)carbonyl) ferrocene dicarboxylate (CD-Fc2-BenC7) was similar to the synthesis of **CD-Fc-BenC7** by using achiral 4-heptylbenzoic acid as the raw material. MALDI-TOF-MS m/z$[M]^+$: 944.37. FT-IR (KBr, cm-1): 2950,

2870 (-CH_3 stretching), 2923, 2853 (-CH_2- stretching), 1735 (-C=OC- stretching), 1608, 1510, 1453 (C=C stretching), 805 (πC-H bending). ^{1}H NMR (500 MHz, $CDCl_3$) δ (ppm) 8.11 (ArH, d, J = 8.9 Hz, 1H), 8.05 – 7.54 (ArH, m, 7H), 7.48 (ArH, dt, J = 9.0, 1.7 Hz, 2H), 7.41 – 7.26 (ArH, m, 4H), 7.26 – 7.14 (ArH, m, 2H), 7.11 – 7.01 (ArH, m, 4H), 4.77 (FcH, dt, J = 2.6, 1.3 Hz, 1H), 4.65 (FcH, ddt, J = 17.7, 2.6, 1.3 Hz, 2H), 4.40 (FcH, dtd, J = 13.8, 2.5, 1.3 Hz, 2H), 4.32 (FcH, dt, J = 2.7, 1.4 Hz , 1H), 3.77 (FcH, td, J = 2.6, 1.3 Hz, 1H), 3.64 (FcH, td, J = 2.6, 1.4 Hz, 1H), 2.59 – 2.43 (Ar-H, m, 3H), 1.89 (-CH_2-,t, J = 12.3 Hz, 5H), 1.56 (-CH_2-, s, 3H), 1.47 (-CH-, dd, J = 12.9, 3.3 Hz, 1H), 1.41 – 1.34 (-CH2 -, m, 2H), 1.35 – 1.19 (-CH_2-, m, 12H), 0.91 (-CH_3, dt, J = 17.6, 7.2 Hz, 6H). Elemental analysis: Calcd. for $C_{61}H_{60}FeO_6$: C, 77.53; H, 6.40; Fe, 5.91; found: C 77.60%, H 6.59%; Fe 5.86%.

2. X-ray photoelectron spectroscopy (XPS)

Furthermore, the Fe valence states of the CD-Fc^+BF4 powder samples before and after oxidation were characterized by XPS within the Fe 2p region. Since the Fe (II) in CD-Fc was oxidized to Fe (III), the characteristic spectral line of Fe (II) disappeared and the characteristic spectral line of Fe (III) appeared in the Fe 2p region. There were the similar results as mentioned above found in all the CD-Fc molecules.

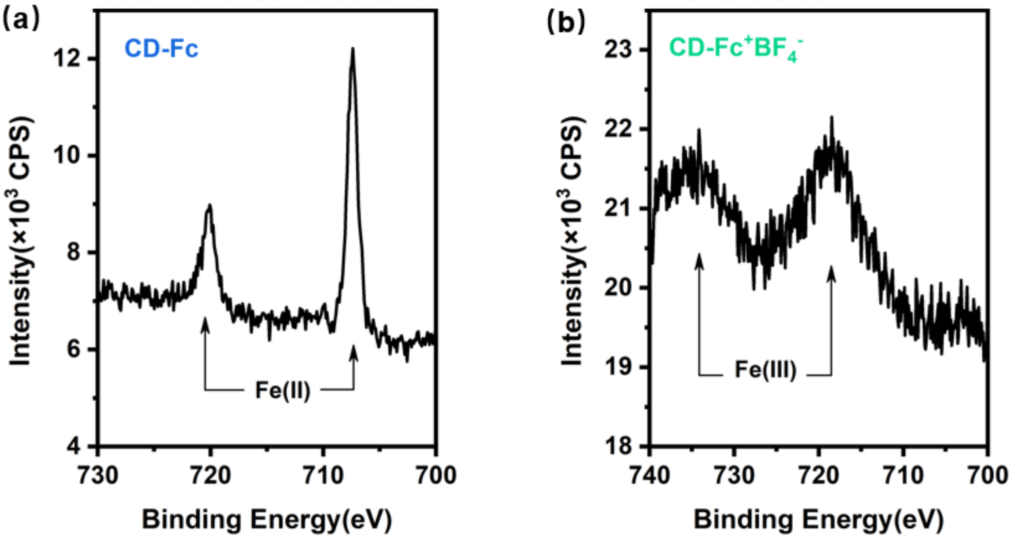

Figure S1. X-ray photoelectron spectra of (a) CD-Fc-C7 and (b) CD-$Fc^+BF_4^-$C7 powder samples in the Fe 2p region.
